# Hospital and Institutional News

**Published:** 1913-10-25

**Authors:** 


### HOSPITAL AND INSTITUTIONAL NEWS.

### THE TREATMENT OF TUBERCULOSIS: HOW THE L.C.C. SCHEME IS PROGRESSING.

Gradually, though not without a certain amount of difficulty, the Public Health Committee of the London County Council is evolving its scheme for the treatment of tuberculosis in the Metropolis. A matter which has aroused a good deal of discussion and correspondence between the Council and the Local Government Board is the question of dispensaries and what body should be responsible for their provision and upkeep. The Local Government Board in their latest letters adhere to the idea that the County Council should be the tuberculosis authority for London, and that through the Council all the various agencies interested in the matter should be co-ordinated so as to form a part of one complete scheme. The Public Health Committee of the Council, however, object that while they have been engaged in the preparation of a comprehensive scheme the Local Government Board have, without reference to the Council, approved local schemes prepared by the local sanitary authorities for the provision of dispensary treatment. The Committee contend that this has rendered it very difficult, if not impossible, for the Council to prepare a comprehensive scheme for London.

### THE BOARD'S ANSWER.

The Board, however, reply that in some cases the urgency of the matter has prevented them consulting the County Council, but in the further development of dispensary schemes the Board will communicate with the Council. The Public Health Committee consider that all local schemes should be submitted to the County Council in the first instance and that, so far as uninsured persons are concerned, contributions in relief of local rates should be made out of the County funds subject to certain safeguards. It is suggested that in cases where the Council disapproves of a local scheme and the local authority concerned refuses to make amendments to meet the Council's objections the Council should make no contribution. It is hoped, in short, that through the medium of finance the Council will be able to secure a measure of control which can now be obtained by no other means. So far no clue is forthcoming as to the cost of the scheme. The Committee confess that at this stage they are unable to put forward even an approximate estimate of the expenditure which will fall upon the county rate. They can only state that, from details which they have before them of the estimated cost of certain local schemes which have been prepared, the annual charge on the county rate may amount to more than ten thousand pounds. In the case of approved schemes of dispensary treatment of uninsured persons 50 per cent. of the expenditure of the Borough Councils will be borne by the Imperial Exchequer, and the proposal of the Public Health Committee is that the County Council shall contribute half of the remainder.

### NATIONAL INSURANCE: DEPOSIT CONTRIBUTORS

As everyone familiar with the National Insurance Act is well aware, insured persons are not compelled to join one or other of the approved societies, but have the option of becoming "deposit" or "Post Office" contributors. As a matter of acknowledged fact, such persons have nothing to gain and a very great deal to lose by adopting this latter course; and, in fact, the Act was expressly drawn so as to discourage people as much as possible from so acting. Deposit contributors are, indeed, not "insured" at all in the proper meaning of the word insurance; and no one who understands his own interests would dream of becoming one of them. In spite of this a small percentage of the insured refuse to join a society and exercise their right to become Post Office contributors. Mr. Masterman has instructed the Chief Inspector under the English Commission to inquire into and report upon the reasons for this obstinacy and perversity; and Mr. Gowers' report to Mr. Masterman is now published by the Stationery Office and the usual Government booksellers. Before we analyse the conclusions at which Mr. Gowers arrives, it may be well to premise that the inclusion of this apparently absurd provision to enable the insured to cut their own throats, so to speak, was due to political motives and a desire to avoid unpopularity of the Act as far as possible.

### THE REASONS FOR THEIR CHOICE.

To test the motives of those who preferred to become deposit contributors an investigation was undertaken of the whole of those who did so in two widely separated and fundamentally unlike communities. One of these was a cathedral city in the West of England, the other a typical Lancashire manufacturing town. The results proved to be closely similar in the two districts. In no fewer than 248 out of the 398 cases (273 men and 125 women) who were interrogated, the adoption of the status of a deposit contributor was attributable wholly to indifference or ignorance. Very few had any wrong-headed idea that they were gaining an advantage, whereas great numbers of them would not bother, or did not care enough, to pay attention to the matter at all. Out of these 248, as a result of the inspector's visit or of the activity of canvassers from the societies, more than 70 per cent. have already decided to join a society and abandon their present status. Of the remaining 150, intermittency of employment, hostility to the Act, ill-health or infirmity, early prospects of escaping from its provisions, and misapprehensions as to the obligations of a member of a society account for the majority. In other words, muddle-headedness is the principal factor in the production of deposit contributors. We note also that one woman who was refused by three societies on account of her impaired health was accepted by a fourth, which shows how blind some society officials are to the prospect of serious inroads on their funds compared with the piling up of imposing aggregates of members.

### LARGE BEQUEST FOR THE MIDDLESEX HOSPITAL.

The late Colonel John Hill, R.E., who lived at Brighton, has bequeathed the residue of his property to the Middlesex Hospital. The entire estate consists of over £31,000 gross, and £29,000 net. The residuary estate now passing to the hospital will be some £25,000 gross, or after payment of legacy duty about £22,000 net. This large bequest, we have no doubt, the Middlesex Hospital will know how to take care of to employ for the best advantage of the sick. Naturally, the first thought of institutional managers will be congratulation of the authorities there on their good fortune. The details of the sums available serve also to show what a huge slice the State takes out of bequests to hospitals; in this case about an eighth of the whole sum. We have remarked before on the injustice and rank ingratitude of this particular impost; and the present circumstances merely serve to confirm us in detestation of it. The Middlesex Hospital, like many others, takes a tremendous financial burden off the shoulders of the State in connection with the working of the Insurance Act; and in return the State exacts an enormous percentage of every sum bequeathed to the hospital to enable it to carry on its work! It is, of course, only a matter of time before reform comes; but that, we fear, is poor consolation for the Middlesex Hospital to-day.

### THE NEW KING'S COLLEGE HOSPITAL.

The new King's College Hospital commenced operations on Monday, October 13, by opening its Casualty Department. In the opinion of Captain Tunnard, the secretary, there has been "a fair demand" on the services of the department, in connection with which two small wards are open to take in serious cases. Apart from these there are no other wards yet available, and no date can at present be given for the reception of in-patients. Some speculation has been aroused in the neighbourhood of Denmark Hill by the erection of a statue, without inscription, in the forecourt of the hospital. A not unreasonable surmise has been that this is the much-delayed monument to the brightest of the luminaries of "King's"—Lord Lister—but in point of fact it is a statue of Dr. Todd, one of the founders of the institution. The statue formerly stood in the old hospital, and it has been given a coat of whitewash to render it in keeping with its new surroundings.

### HOSPITAL SATURDAY IN LONDON.

Although it is too soon to announce the result of the appeal of the Lord Mayor of London (Sir D. Burnett), the President, on behalf of the annual Hospital Saturday Collection of the 11th inst., and that of the Chairman (Sir Savile Crossley, Bart.) in support especially of family collections as a means of compensating for the shrinkage of income which has been evinced this year, yet it appears from inquiry made at the Central Office, Gray's Inn Road, that some of the local committees have had very encouraging results and will have larger totals than hitherto. The Fund, including past results of the annual collection, reached down to Saturday last about £16,000. This total is, of course, exclusive of several thousands of pounds received in part payment of benefits, which will ultimately be added to the total of the ordinary receipts for distribution among the medical charities. The weekly collection—the main source of the income—will be continued until the end of the year. Among the collections made in places of amusement on Hospital Saturday were: Lyceum Theatre, £11 17s. 9d.; Wimbledon Theatre, £20 19s. 8d.; Oxford Music Hall, £15 8s. 3d.; and Daly's Theatre, £5 0s. 3d.

### NELSON HOSPITAL FOR WIMBLEDON AND MERTON.

The Nelson Hospital is fortunate in possessing the good will of the cricketers and football players of the neighbourhood. Friends connected with clubs in the western part of the district have just offered to guarantee a sum of £50 annually in support of a bed in the adult male ward, and this welcome proposal follows hard upon aid received through a cricket match specially organised on behalf of the Nelson Hospital and the North Wimbledon Cottage Hospital, and played on Saturday, September 27. As a result of this effort cheques for £105 have already been received by each hospital, and there is said to be some £60 yet to be divided. The teams competing were a Wimbledon Town team and an eleven captained by Mr. J. B. Hobbs, the well-known Surrey professional. The match created extraordinary enthusiasm, and there was an enormous attendance. A bat, inscribed with names of the players, realised £86, and the efforts of a troupe of pierrots, who were at work all day and until nearly midnight, added £20 to the total. The two hospitals will also probably benefit to the extent of £350 each in the money, collected under the auspices of the Mayor and other prominent Wimbledonians, for a memorial to the late Rev. Father Kerr, each hospital undertaking to name a cot after the deceased gentleman.

### A MOTOR-'BUS AMBULANCE.

Tenders recently invited and received by the Westminster Guardians for a ten-passenger motor omnibus, which can be converted into a two-stretcher ambulance van, are of distinct interest as a lead to the London County Council in the matter of street ambulances. Twelve tenders, it is stated, were opened, and they ranged from £485 to £762. As there were two tenders at the lowest price, it was decided to refer for expert advice concerning them. We may suggest that expert advice would surely be preferable about the whole set, not about the two cheapest alone: there is such a thing as quality about motor vehicles as about other things. But we dwell upon this new idea more particularly because the County Council, which has been so reluctant to shoulder its responsibilities in providing street ambulances, may take to heart the action of the Westminster Guardians. The Council possesses any number of motor-'buses for taking crippled children to school; why should not some of these be made convertible into ambulances and available as such when not wanted for the children?

### LONDON AND ITS AMBULANCES.

There seems at last to be some prospect of the London County Council proceeding with its ideal for improving the ambulance service of the Metropolis. At a meeting of the Council on October 14, Major Gray, chairman of the General Purposes Committee, which has the matter in hand, said that Parliament had now given them the powers which they sought whereby they might co-ordinate the ambulance service of London. Under those powers he had no doubt that they would be able to provide an effective and efficient ambulance service for the whole of London, and that he trusted before the close of the current year.

### MEDICAL AND DENTAL TREATMENT OF SCHOLARS.

The Board of Education has acquiesced in the proposals of the London County Council for the medical and dental treatment of school children during the ensuing year. Agreements with certain hospitals and school treatment centres are to be renewed and provision made for further medical dental and nursing treatment. One feature of the new scheme is the reduction of the numbers to be treated at each centre; but this is compensated for by the increase in the number of centres. The Board of Education has intimated that it will not raise any objection to the proposal to undertake the treatment by $x$-rays at the Fulham School Centre of children suffering from ringworm. The L.C.C. has also had under consideration the need for employing an officer in connection with school children attending Guy's Hospital for treatment. No arrangement has been made under the Council's scheme with the authorities of the hospital, but as a large number of children attend the hospital the Council is of opinion that an assistant organiser with nursing qualifications should be engaged to assist at the hospital and to follow up the cases of children attending for treatment. The hospital authorities concur in this suggestion, and the Council accordingly proposes to appoint such an organiser at a salary of £2 a week.

### THE PROVISION OF X-RAY DEPARTMENTS.

All over the country hospitals of all sorts and sizes are confronted daily with the urgent necessity of establishing $x$-ray and electro-therapeutic departments for the many new kinds of treatment which can be carried out only by means of such special departments. At many places the considerable expense thus entailed has already been faced and modern apparatus provided; and many of those institutions which have not yet done the same are earnestly considering ways and means. At Cromer this difficulty is in a fair way to be solved in a particularly graceful way. Mr. Dent, who has since his retirement from the Army Medical Service practised there for a quarter of a century, is about to retire. His numerous friends desired to mark their appreciation of his work, both in practice and at the Cromer Cottage Hospital, by a presentation; and Mr. Dent was approached upon the subject. He expressed, however, an earnest desire that any sum collected for this purpose should be handed not to him, but to the Cottage Hospital. Accordingly, Mr. Locker-Lampson has issued an appeal for such a sum (some £300 to £400) as will enable a complete $x$-ray apparatus to be provided for the Cottage Hospital, and this happy thought will surely meet with a hearty response. Mr. Dent's peculiarly gracious renunciation of any presentation to himself, in favour of the hospital for which he has already laboured so abundantly, is an incident which should certainly quicken the sympathy of the people of Cromer in the matter of this proposed augmentation of their Cottage Hospital's potential usefulness.

### NEW CHILDREN'S HOSPITAL FOR ABERDEEN.

A site has been secured at Aberdeen for the proposed children's hospital in that city, a project which has the approval and the warm personal interest of Her Majesty the Queen. The situation and size of the plot of ground which has been secured for the hospital are said to be excellently suited for the purpose in view. Financially the movement is making rapid progress, though it has not yet got completely on its legs. The total amount required for the building fund is £30,000, of which just over £20,000 has been secured; the largest donation so far received has been £5,000 from Lord and Lady Cowdray. It is felt that the interest and appreciation displayed by the Queen at a recent visit to Aberdeen are certain to help forward the building scheme. The Girls' High School held a *café chantant* recently on behalf of the building fund, and a net profit of over £75 was realised. Furthermore, Mrs. Maitland, the wife of the Lord Provost, is organising a Town Council Ladies' Committee, which has just begun work and has already collected £15; 440 coupon-books, each containing forty sixpenny coupons, have already been issued, and care is being taken to interest districts outside Aberdeen from which patients are likely to be drawn. So much has now been accomplished towards the fruition of this scheme that we cannot doubt its speedy realisation; and it is much to be desired that the new hospital should be opened entirely free from debt, and indeed preferably with a substantial sum in hand as the nucleus of an endowment.

### HOSPITAL LOSSES THROUGH INSURANCE ACT.

Consequent on the operations of the National Insurance Act, a report presented by the Honorary Treasurer at the last quarterly meeting of the King Edward Memorial Hospital, at Ealing, is anything but satisfactory. The cost of drugs had increased as compared with the same period last year, but the receipts from the dispensary dropped £250. So many persons who were paying into the dispensary last year had now withdrawn, as they were insured, and were only interested in it so far as their wives and families were concerned. Moreover, the hospital was losing a large number of subscribers, who expressed the view that there was now no need to subscribe since the Insurance Act had come into force. Alderman J. Box, J.P. (chairman), remarked that hitherto the committee had been able to make a charge to masters and mistresses for their servants, but the servants, being insured, many now decline to pay anything. It was undoubtedly a very big question, which they would have to tackle before long. Observing that they had been desired by the British Medical Association not to make any definite arrangements at present regarding insured persons, Alderman Box said they had had this year to admit to hospital many insured cases, which were really destitute cases, but the Poor-Law Infirmary at Isleworth refused them, saying, of course, no person could be destitute when in receipt of 10s. a week!

### MASONIC NURSING HOMES.

An important announcement is that of the approaching maturity of a scheme for the establishment of contributory self-supporting Nursing Homes for Freemasons. A general committee to deal with the fructification of a three-years-old project has been formed, and has held its first meeting under the chairmanship of Lord Ampthill. Mr. C. E. Keyser was appointed chairman for the future work of the committee, and various donations and promises of support were announced. Twenty of the provinces into which British Freemasons are organised have already agreed to support the scheme, and the remainder will be approached. It will be of interest to note in due time what measure of success attends this ambitious and philanthropic enterprise, to which we wish in anticipation every possible success. The list of leading Freemasons who have already become patrons of the scheme contains many well-known and highly respected names, including those of a number of Provincial Grand Masters.

### FIRST AID FOR HOSPITAL PORTERS.

An incident which recently occurred at King Edward VII.'s Hospital, Cardiff, and its subsequent discussion by the board of management, have served to ventilate a matter which seldom receives the attention to which it is on its merits entitled—namely, the training of hospital porters in first-aid and ambulance work. It seems that complaints were made concerning the way in which a certain patient was transferred from a stretcher to a bed at the hospital; in fact, the allegation is that the patient was tumbled off the stretcher on to the bed, and it is also stated that this charge was confirmed by the testimony of the medical officer. The particular point concerning any individual case was not pressed; but Mr. William Thomas proposed at the board meeting a resolution to the effect that all porters employed at the hospital should be trained to stretcher work. Colonel Bruce Vaughan did not think the resolution necessary, and upheld the porters against the attacks made upon their skill. He did not, however, make out a very strong case, for his best argument seemed to be that the porters learn how to handle patients by virtue of the great practice which they obtain. In other words, they pick it up by experimental methods upon the patients, according to Colonel Vaughan. After this, it is not surprising to note that the Chairman, to get once more in touch with the general sense of the meeting, had to change his tone and to raise no objection to the resolution being accepted and the matter being referred to the house committee, which was accordingly done. This question of the training of hospital porters is one with even wider applications than those raised at Cardiff, and represents one of the minor problems of hospital administration which are not as yet solved by general consent among institutional authorities.

### INCORPORATED ASSOCIATION OF HOSPITAL OFFICERS.

Mr. Herbert H. Jennings, secretary of the Chelsea Hospital for Women, has been elected president of the Incorporated Association of Hospital Officers for the Session 1913-1914. Mr. Jennings has been closely connected with the Association almost from its inception, having been honorary treasurer for a number of years, and latterly chairman of the council. The new chairman is Mr. Godfrey H. Hamilton, secretary of the National Hospital for the Paralysed and Epileptic, who recently made an unwelcome acquaintance with a police-court in connection with a contravention of the Cinematograph Act. The only other change of note amongst the officers of the Association is in the honorary assistant-editorship of the *Hospital Gazette*, which Mr. F. P. Carroll has been compelled to relinquish on account of the pressure of other work. This vacancy also has been filled by the appointment of Mr. Godfrey Hamilton, who will thus act in a dual capacity. The programme of meetings for the coming session contain several noteworthy items, amongst them being a paper on "Suggestions for Adapting the Existing Voluntary System to Meet Present Requirements," by Mr. H. W. Burleigh; and a joint conference with the Poor-Law Officers' Association. In addition many papers of a practical character will be read, and a number of visits paid to other hospitals.

### PRESENTATION TO DR. C. H. WISE.

During the past week numerous presentations have been made to Dr. C. H. Wise on his leaving Walthamstow (his resignation of the post of senior hon. medical officer to the Walthamstow Hospital was noted in these columns last week). The Committee and Staff of the hospital presented him with an invalid-chair, a reading-table, a reading-lamp, and a case of cutlery; and the Walthamstow Medical Society showed their appreciation by the gift of a solid silver fruit-dish. At a recent meeting of the Council of the Walthamstow Hospital the following resolution was passed: "That the Council of the Walthamstow, Leyton, and Wanstead

Children's and General Hospital received with the greatest regret the resignation of Dr. C. H. Wise as the senior Hon. Medical Officer. Dr. Wise has since the foundation of the Hospital so generously placed his almost daily services at its disposal, and by his skill and judgment has helped to such a very large extent to maintain its efficiency, that the Council desire to express their sincere thanks for what he has done, and to say how much they regret that ill-health is the cause of his removing from the neighbourhood."

### THE GLASGOW MATERNITY HOSPITAL.

The new buildings of the Glasgow Maternity and Women's Hospital were opened in 1908, and excellently equipped gynæcological wards were added in 1911. Unfortunately, the responses to building fund appeals have fallen short of the expenditure by no less than £28,000, which large debt is not yet liquidated. Further, the support of the public for the annual upkeep has not been by any means liberal; and the directors have decided to close the gynæcological wards until the financial position is more satisfactory. This decision on the face of it seems inevitable; and, indeed, should probably have been arrived at some time ago. A total sum of £110,000 was spent on the buildings, of which nearly three-quarters has been raised; so that local effort has certainly been creditable, even if insufficient. It is the deficiency of income from annual subscriptions which is the really serious matter; and in view of the work amongst the women of Glasgow which the hospital does, it is greatly to be hoped that the closing will be only temporary. The Duke and Duchess of Argyll are giving their patronage to a bazaar which is being organised on a very elaborate scale with a view to reducing the debt. Bazaars are hateful expedients, but if ever one was admissible and legitimate, we should think this one is; and we hope a large total will be realised.

### ANOTHER SCOTTISH WOMEN'S HOSPITAL.

For seventeen years the Dundee Private Hospital for Women has done good and unostentatious work, and the Society which manages it is on the eve, we learn, of a great expansion of its beneficent operations. A new hospital is being built to accommodate twenty or more patients, and is expected to be completed in a few months; and to indicate this opening of a new chapter in its history the title of the hospital is to be changed to the Dundee Women's Hospital and Nursing Home. The annual meeting of subscribers, recently held, was thus the last of the old series, and was also signalised by the loss of two of the institution's oldest and best friends. Mrs. R. B. Don, the president, has felt obliged to resign, to the great regret of all those interested in the hospital; and Miss Lily Walker, honorary secretary to the hospital from its commencement, has been removed by death. Mrs. Thomas Maitland was requested to accept the vacant presidency, but asked for time to consider the matter. We are glad to note that this charity is partly self-supporting; so that it belongs to that none too numerous class which helps those who help themselves. Its prospective expansion is therefore eminently matter for satisfaction.

### A NORTH BERWICK INSTITUTION.

Under the will of a Miss Edington, who died nearly five years ago, a sum of ten thousand pounds, together with her residual estate, became available for erecting and maintaining a convalescent home. The trust disposition further directed that the home should contain an accident ward and a ward for cases of illness, not being infectious or incurable. The institution is thus seen to partake partly of the character of a small hospital as well as of that of a purely convalescent home. The buildings are now completed; they have a frontage of over one hundred feet, and comprise a main front block, two lavatory wings, and a kitchen block. The accommodation includes five wards, operating theatre, administrative offices, and accommodation for matron, nurses, and servants. The building and furnishing have cost £3,500, so that a quite considerable amount remains in hand for an endowment fund. The Edington Home should prove a great boon to the inhabitants of North Berwick, and has doubtless a future of abundant well-doing before it.

### BIG LEGACY FOR THE STRATFORD-ON-AVON HOSPITAL.

At the annual Court of Governors of this hospital a deficit on the year's working was shown amounting to £245, and the usual tale was told of ever-increasing demands upon the resources of the charity. After some excellently plain speech from Lord Willoughby de Broke on the subject of the support of voluntary hospitals, a very welcome piece of intelligence was communicated by the Rev. F. H. Hodgson, who said he was in a position to assure the court that under a will not yet proved the hospital will shortly benefit to the extent of about £8,000. The income from this splendid legacy will certainly lessen, though it will not abolish, the anxieties of the governors about ways and means. We trust it will not lead to any slackening off of endeavours to obtain new subscribers or to tap new sources of income. A single legacy of this magnitude makes but little difference to the finances of a big urban hospital; but to institutions of the type of that at Stratford-on-Avon it is, of course, a large sum and a very welcome addition to the invested resources.

### HOSPITAL SATURDAY AT BIRMINGHAM.

Always to the front in matters of local patriotism and generosity, the citizens of Birmingham have done well indeed in securing this year over £22,000 by means of their Hospital Saturday Fund. As usual, £10,000 was distributed among the voluntary hospitals of the city and neighbourhood. The rest is spent on convalescent homes, sanatoria, surgical aid, and other forms of relief whose management is retained by the Hospital Saturday Committee. The principal sums allotted out of the £10,000 for voluntary charities were: The General Hospital, £3,150; the Queen's Hospital, £2,100; the General Dispensary, £1,010; the Children's Hospital, £800; the Women's Hospital, £500; the Eye Hospital, £350; the Orthopædic Hospital, £300; and the Jaffray Suburban Branch Hospital, £400. Smaller sums were apportioned to other hospitals and to a number of nursing societies.

### THE EAST SUSSEX HOSPITAL SITE.

Differences of opinion, even heartburnings, have arisen in Hastings and St. Leonards over certain business transactions connected with the sale of the site of the East Sussex Hospital to the Corporation. The purchase price itself is not regarded as excessive, except by a small and unimportant section; but objection has been aroused by the arrangement under which the Corporation pays about fifteen thousand pounds down for the site, whereas possession is not to be yielded for another two years, during which the hospital pays the Corporation the nominal rent of £25. In other words, the hospital gets the use of the large sum of money in question free of interest for two years. The hospital argues that it will take this length of time to rebuild on another site, that the old hospital must be kept open until the new one is ready, and that the fifteen thousand is wanted to get on with the work. A suggestion has been put forward in the local press that the hospital should accept five thousand pounds down, and the balance in two annual instalments of the same amount each. If this arrangement will enable the hospital managers to meet their builders' bills as they fall due, we think as an act of grace they would be wise to accept it or some similar compromise. But we really cannot sympathise with those of the local residents who are actually seeking to go back on the contract. In undertaking to pay cash for a site, possession of which they knew could not be gained for two years, the Corporation were making a bargain with their eyes open, and should not begin to whine about it now. Quite conceivably the hospital managers may say their price would have been larger if the purchase money were not to be paid until two years have passed. Business is business, though it is certainly regrettable if either side to a "deal" afterwards fancies itself harshly or unconscionably used; and it is particularly desirable that voluntary institutions for the sick should afford no grounds for any such feelings of soreness.

### DEATH OF SIR JOHN TUKE.

It is very curious that certain families devote themselves generation after generation to the study of mental diseases. We do not know of any other department of medical science in which such atavism is evident, but among alienists it seems to be very common. Sir John Batty Tuke, M.D., who died on October 13 at Edinburgh, was a conspicuous example of this rule; for his family had been associated almost from time immemorial with the study and treatment of insanity. Curiously enough (for the name is a somewhat unusual one) he was not related, we believe, or at least not nearly related, to the well-known family of the same name who manage a famous private asylum at Chiswick. Sir John Tuke was a Yorkshireman by birth, and married a sister of one of the Archbishops of York (Magee); but he provided one of those very rare instances (Lister was another such case) of an Englishman who attained high medical distinction in Scotland. Nay, more, he even sat for a Scottish constituency in Parliament, for he represented Edinburgh and St. Andrews Universities as a Unionist from 1900 to 1910. At the time of his death Sir John Tuke had attained the age of seventy-eight years. He saw active service in the Maori War of 1860.

### AN INSTITUTIONAL PIGGERY.

A very practical, though somewhat novel, method of keeping down the housekeeping expenses is in vogue at the Bristol Mental Asylum, where a large number of pigs—sometimes as many as 200—are kept on the institution's land, which also is responsible for the main supply of fruit and vegetables. When it is stated that there are some 600 inmates of both sexes and a large resident staff the value of a large piggery is obvious, though, of course, such an industry is only possible for those institutions which have a large amount of land at their disposal.

### REIGATE AND REDHILL HOSPITAL.

The Reigate and Redhill Hospital is to be congratulated in having been freed from their debt of £1,000, which satisfactory state is due to the enthusiasm evoked by the suggestion of a Hospital Saturday in the Borough. When the scheme was suggested to clear the debt a few weeks back, it was well taken up by the Working Men's Committee and the local ladies, though their task was a large one. An appeal was issued, and an anonymous gift of £500 was the result. The addition of other donations left £380 to be cleared; accordingly a street collection was arranged, and the promoters, following the lines of the Alexandra Day Committee, purchased some 20,000 roses from that body, which, as the result of a hard day's work, brought in about £356. This, together with a few additional subscriptions, has cleared the hospital from the burden of debt.

### THIS WEEK'S DRUG MARKET.

Quiet conditions continue to prevail in the Drug market, the expected improvement being slow in making its appearance. The most interesting price change of the week has been a reduction in quotations for cocaine; this announcement will not come as a surprise to those who have followed the market reports. All the makers of strychnine have reduced their quotations. There has also been a reduction in the price of acetyl salicylic acid. There has been a further reduction in the value of oil of cloves. No improvement in the demand for cod-liver oil is noticeable. The price of essence of lemons has a downward tendency. There is a fairly steady tone in the Opium market, but morphine is barely steady. Menthol now has an upward tendency in price. At the recent public sale of drugs in Mincing Lane the general tendency of prices was in a downward direction, but there were several exceptions.

